# Commentary on Booth *et al*.: Measuring the health burden of homelessness

**DOI:** 10.1111/add.16415

**Published:** 2023-12-17

**Authors:** Jessica A. Heerde, Lucas Calais‐Ferreira, Susan M. Sawyer

**Affiliations:** ^1^ Department of Paediatrics The University of Melbourne Parkville Victoria Australia; ^2^ Centre for Adolescent Health Royal Children's Hospital Parkville Victoria Australia; ^3^ Murdoch Children's Research Institute Parkville Victoria Australia; ^4^ Department of Social Work The University of Melbourne Parkville Victoria Australia; ^5^ Centre for Mental Health, Melbourne School of Population and Global Health The University of Melbourne Parkville Victoria Australia; ^6^ Justice Health Group, School of Population Health Curtin University Perth Western Australia Australia

**Keywords:** Adolescents, data linkage, health, health‐care, homelessness, substance use and misuse

*Substance use and misuse are significant health issues for homeless people, including adolescents. Lack of robust research means that we risk underestimating the extent to which homelessness drives health risk throughout the life‐course. Linked administrative data hold untapped opportunities to understand and respond to the largely preventable health burden of homelessness*.

At the end of the first quarter of the 21st century, rising living costs and inflation are having flow‐on effects on the cost of food, housing and other essential commodities, increasing risks for homelessness. In many countries and regions, climate‐driven displacement, family dislocation due to war, political conflict and migration are also heightening risks. These threats are further compounded by associated unemployment and job loss, family violence and relationship breakdowns, which are known contributors to homelessness [1]. Notwithstanding the extent that social determinants drive health outcomes throughout the life‐course, the extent to which homelessness is associated with early death and other preventable morbidities, including substance use and misuse, has not been well‐characterized.

In this context, the study by Booth and colleagues [2] makes an important contribution. The opioid epidemic is a public health issue of international concern, particularly in North America, where a disproportionately high number of young and middle‐aged adults have died [3]. The study reports opioid‐related mortality among people experiencing homelessness in Canada using linked administrative data sources. The authors analysed retrospective data from 6644 individuals aged 18–65+ years who died of accidental opioid‐related overdose from 2017 to 2021. They found a considerably higher rate of death in homeless people (one in six, where homelessness was determined by the Coroner or recorded as part of a health‐care visit within the year preceding their death) compared to the general population (one in 150). Those experiencing homelessness were younger (aged 25–44 years) than the general population, and 7% of those who died were aged 18–24 years. Unfortunately, the study did not extend to adolescents aged less than 18 years. This is disappointing, because while adolescents who experience homelessness are also likely to face a high risk of early preventable death [4], they may be more amenable to social, education, housing and health interventions due to less severe or entrenched comorbidities when compared to older adults.

A quick search of papers published in *Addiction* over the past 20 years reveals few papers (*n* = 13) focused upon addiction and substance misuse among people experiencing homelessness. Of these, only two focused upon adolescents [5, 6]. This low number of papers probably reflects the multiple challenges that researchers face in conducting robust research with homeless people. In adolescents, existing studies have been limited by poor data quality, limited measures of health outcomes and difficulties in sampling, engaging and tracing homeless adolescents in traditional observational or intervention studies [4]. For example, most studies have analysed data from small or highly selected samples of adolescents experiencing homelessness without a comparison group of adolescents who were not homeless [1]. The result of these limitations is that homelessness research has historically been poor in quality. Consequently, in failing to fully understand the health needs and outcomes of adolescents experiencing homelessness, we probably underestimate the extent to which homelessness drives health risks throughout the life‐course.

For this reason, there is a strong case for broadening the scope and design of research on the health of adolescents experiencing homelessness. As shown by Booth and colleagues [2], this opportunity exists through using multi‐sectoral linkage of administrative homelessness and health data, including substance use. Multi‐sectoral data linkage provides new opportunities for characterizing the fatal and non‐fatal burden from substance use among homeless adolescents, pathways through health‐care (including emergency departments and substance use treatment services) and coordination and continuity of care among health settings. Routine monitoring of these data can identify emergent health‐care needs (e.g. increases in infectious diseases or overdoses), help to inform the allocation or reallocation of health resources and potentially modify existing models of health‐care. Substance misuse in adolescents experiencing homelessness is highly comorbid with other health issues (e.g. mental health disorders, including developmental disorders and trauma and communicable diseases), violence, victimization and exploitation [7, 8]. Multi‐sectorial data linkage also offers specific advantages for studying the health and health‐care trajectories of homeless adolescents who have been exposed to other compounding social determinants, such as youth justice involvement [9, 10], child protective services [11, 12] or those who use illegal substances, which typically challenge ethics committees.

The successful transition to adulthood of the world's 1.8 billion adolescents sets the foundation upon which future population health is shaped [13, 14], but the many challenges facing contemporary families, children and adolescents results in growing vulnerability to homelessness. Multi‐sectoral data linkage holds untapped opportunities to fill important data gaps, including around the complex intersections of homelessness and substance use experienced by adolescents as well as adults.

## AUTHOR CONTRIBUTIONS

**Jessica A. Heerde:** Conceptualization (lead); writing—original draft (lead); writing—review and editing (lead). **Lucas Calais‐Ferreira:** Writing—original draft (supporting); writing—review and editing (supporting). **Susan M. Sawyer:** Writing—original draft (supporting); writing—review and editing (supporting).

## DECLARATION OF INTERESTS

None to declare.
